# Differentiation Behaviour of Adipose-Derived Stromal Cells (ASCs) Seeded on Polyurethane-Fibrin Scaffolds In Vitro and In Vivo

**DOI:** 10.3390/biomedicines9080982

**Published:** 2021-08-09

**Authors:** Katrin Radeloff, Dorothee Weiss, Rudolf Hagen, Norbert Kleinsasser, Andreas Radeloff

**Affiliations:** 1 Department of Otorhinolaryngology, Head and Neck Surgery, Evangelisches Krankenhaus, Carl von Ossietzky-University of Oldenburg, 26122 Oldenburg, Germany; andreas.radeloff@uni-oldenburg.de; 2 Department of Otorhinolaryngology, Plastic, Aesthetic and Reconstructive Head and Neck Surgery, Julius-Maximilian-University of Wuerzburg, 97080 Wuerzburg, Germany; doro_weiss@gmx.de (D.W.); hagen_r@ukw.de (R.H.); kleinsasse_n@ukw.de (N.K.)

**Keywords:** polyurethane, fibrin, ASC, adipose-derived stromal cells, chondrogenic differentiation, endochondral ossification, BMP-6, TGF-ß3

## Abstract

Adipose-derived stromal cells (ASCs) are a promising cell source for tissue engineering and regenerative medicine approaches for cartilage replacement. For chondrogenic differentiation, human (h)ASCs were seeded on three-dimensional polyurethane (PU) fibrin composites and induced with a chondrogenic differentiation medium containing TGF-ß3, BMP-6, and IGF-1 in various combinations. In addition, in vitro predifferentiated cell-seeded constructs were implanted into auricular cartilage defects of New Zealand White Rabbits for 4 and 12 weeks. Histological, immunohistochemical, and RT-PCR analyses were performed on the constructs maintained in vitro to determine extracellular matrix (ECM) deposition and expression of specific cartilage markers. Chondrogenic differentiated constructs showed a uniform distribution of cells and ECM proteins. RT-PCR showed increased gene expression of collagen II, collagen X, and aggrecan and nearly stable expression of SOX-9 and collagen I. Rabbit (r)ASC-seeded PU-fibrin composites implanted in ear cartilage defects of New Zealand White Rabbits showed deposition of ECM with structures resembling cartilage lacunae by Alcian blue staining. However, extracellular calcium deposition became detectable over the course of 12 weeks. RT-PCR showed evidence of endochondral ossification during the time course with the expression of specific marker genes (collagen X and RUNX-2). In conclusion, hASCs show chondrogenic differentiation capacity in vitro with the expression of specific marker genes and deposition of cartilage-specific ECM proteins. After implantation of predifferentiated rASC-seeded PU-fibrin scaffolds into a cartilage defect, the constructs undergo the route of endochondral ossification.

## 1. Introduction

Cartilage defects of the head and neck region are due to trauma, infections, congenital malformations, or surgical procedures, e.g., for malignancies. Common therapeutic methods include the use of autologous cartilage from other locations, such as rib cartilage for the reconstruction of the auricle [1] or auricular cartilage for closure of nasal septal defects [2]. Complex reconstruction procedures of laryngotracheal defects, for instance, include the transplantation of autologous cartilage tissue combined with local or microvascular flaps [3]. Nowadays, tissue engineering and regenerative medicine approaches may be promising methods for replacing cartilage tissue of the larynx and trachea, auricle, or nose [4,5,6,7,8,9].

Although the use of chondrocytes for cartilage tissue engineering has been extensively studied and transferred to clinical applications [6,10], many groups have focused on mesenchymal stem cells (MSCs) from bone marrow (BMSCs) [11] or adipose tissue (ASCs) in their studies [12]. ASCs can easily be obtained from, e.g., liposuction material by enzymatic or mechanical digestion of the lipoaspirate. The donor-site morbidity in adipose tissue harvesting is low [13,14], and the cell yield is very high [13,15,16,17]. ASCs share immunophenotype characteristics with BMSCs, including their ability of multilineage differentiation and the paracrine secretion of cytokines and growth factors [17,18,19,20,21,22,23]. 

ASCs have the potential to undergo chondrogenic differentiation stimulated by defined chondrogenic differentiation media containing growth factors such as members of the transforming growth factor beta (TGF-ß) family, including TGF-ß1 [21,24], TGF-ß3 [24,25], and bone morphogenic protein (BMP)-6 [24,25]. However, the capacity of ASCs to differentiate into the chondrogenic lineage is lower than that of BMSCs [25,26,27]. Experiments by our own group with the induction of chondrogenic differentiation of ASCs in PU-fibrin composites using various single growth factors revealed a low tendency of ASCs to undergo chondrogenesis [28]. This limited chondrogenic potential of ASCs may be due to reduced expression of the TGF-ß receptor [25] and cell surface marker vascular cell adhesion molecule 1 (CD106) [29]. Thus, in the literature, considerations have been described to overcome this limitation [25,27,30,31]. A more effective stimulation towards chondrogenic differentiation of ASCs similar to BMSCs has been reported to be obtained by, e.g., high doses of BMP-6 [27,29], high concentrations of TGF-ß and IGF [27,32], and the combination of TGF-ß3 with BMP-6 [25,27,30,31]. 

In addition, co-culture of ASCs and chondrocytes has been reported to be promising in the generation of cartilage tissue [24,33], and it seems very important to keep the MSCs in a three-dimensional culture system, such as a micromass pellet or a three-dimensional scaffold [34]. 

Polycaprolactone-based polyurethane (PU) scaffolds offer a three-dimensional structure and can be produced in various shapes with high porosity, specific hydrophilicity, and a reasonable degradation time. Furthermore, they are volume-stable and mechanically resilient, a property that is beneficial for in vivo implantation and prevents deformation of the construct [35]. However, a three-dimensional, even cell distribution is not guaranteed because of the two-dimensional cell seeding on the pore walls with subsequent cell dedifferentiation [36]. In addition, poor retention of synthesised extracellular matrix (ECM) molecules and diffusion of ECM proteins into the culture medium have been described [36,37,38]. Hydrogels such as fibrin gel can have ECM-like properties, ensure an even cell distribution and three-dimensional retention of ECM due to a narrow fibre network. This eliminates the disadvantages of solid scaffolds like PU [35,36,38]. The combination of long-stable fibrin gel and the PU scaffold has been reported to obtain an even cell distribution and sufficient retention of extracellular matrix proteins in a volume-stable construct [35,36,38]. 

The present study consists of two parts: in vitro, certain growth factor combinations for chondrogenic differentiation of hASCs seeded in PU-fibrin scaffolds were compared. In order to evaluate their behaviour in vivo, PU-fibrin scaffolds were seeded with autologous ASCs of New Zealand White rabbits and implanted into auricular cartilage defects after precultivation in vitro.

## 2. Materials and Methods

### 2.1. In Vitro Studies

#### 2.1.1. Isolation and Culture of Human Adipose-Derived Stem Cells (hASCs)

Human ASCs were isolated as described before [28,39,40] from the subcutaneous adipose tissue of healthy female donors (n = 4) after obtaining their informed consent. The study was approved by the Institutional Review Board of the Medical Department of the Julius-Maximilian University of Wuerzburg (grant #16/06, 25 July 2008).

The lipoaspirate was washed with phosphate-buffered saline (PBS; Roche Diagnostics, Mannheim, Germany) plus 1% penicillin/streptomycin (P/S; Biochrom AG, Berlin, Germany). Collagenase P solution containing 10 mg Collagenase P (Roche Diagnostics, Mannheim, Germany) in PBS per 100 mL lipoaspirate material was used for the digestion procedure at 37 °C under continuous shaking. Subsequently, the pelleted stromal vascular fraction (SVF) was separated from the adipocyte fraction by centrifugation. After discarding the oily layer, erythrocytes in the remaining cell pellet were eliminated by incubation with erythrocyte lysis buffer (154 mM ammonium chloride (NH_4_Cl; Sigma-Aldrich, Steinheim, Germany), 10 mM potassium bicarbonate (KHCO_3_; Sigma-Aldrich Steinheim, Germany), and 0.1 mM ethylenediaminetetraacetic acid (EDTA; Sigma-Aldrich Steinheim, Germany). After a further centrifugation and washing step, cells were resuspended in an expansion medium consisting of Dulbecco’s modified Eagle’s medium (DMEM; Gibco Invitrogen, Karlsruhe, Germany) plus 1% P/S and 10% fetal calf serum (FCS; Linaris, Wertheim-Bettingen, Germany) (EM-DMEM) and filtered with a 100 μm cell strainer (BD Bioscience, Bedford, MA, USA) to remove the remaining soft tissue. The cells were plated in culture flasks at a density equivalent to approximately 0.2 mL of liposuction tissue aspirate/cm^2^ of surface area and maintained at 37 °C in a humidified atmosphere and 5% CO_2_ for expansion and culture. These primary cells were defined as passage 0 (P0) cells. During expansion, the medium was replaced every 3 days. When the cells reached 80% confluency, they were detached with 0.25% trypsin containing 1 mM EDTA (Gibco Invitrogen). Subsequently, 1 × 10^6^ ASCs were resuspended in 1 mL cryopreservation medium (80% FCS, 10% DMEM and 10% dimethylsulfoxide (DMSO (Sigma-Aldrich, Steinheim, Germany)), frozen at −80 °C in an ethanol-jacketed closed container for 2 days and afterwards stored in liquid nitrogen. For the following experiments, hASCs were rapidly thawed and centrifuged to remove DMSO. Subsequently, ASCs were resuspended in EM-DMEM, seeded again in culture flasks, and maintained at 37 °C and 5% CO_2_ for proliferation. When the cells reached 80% confluency, they were detached and transferred into new flasks for the next passage. ASCs of passage 3 were used for the following experiments. Following the position paper of Bourin et al. [22], ASCs were verified by flow cytometric analysis of specific cell surface markers and multilineage differentiation procedures (data not shown).

#### 2.1.2. Fabrication of Polyurethane-Fibrin Composites 

Polyurethane (PU) foam was kindly provided by Polymaterials (Kaufbeuren, Germany) as discs with a diameter of 7 cm. PU scaffolds were prepared by punching discs of 5 × 2 mm using a biopsy punch (∅ 5 mm, Harris Uni-Core™, Redding, CA, USA). Several washing steps using 70% ethanol and PBS were performed before the small polyurethane disks were autoclaved in a flask containing 50 mL PBS. Aprotinin solution (Trasylol^®^) was purchased from Bayer (Leverkusen, Germany), and the fibrin glue kit Tissucol^®^, including thrombin and dilution buffer, was obtained from Baxter (Unterschleißheim, Germany). An amount of 100 mg of fibrinogen (Sigma-Aldrich, Steinheim, Germany) was mixed with 3000 KIE/mL aprotinin solution and sterile filtered. Thrombin solution was prepared by dissolving 500 IE thrombin in 1 mL of 40 mM calcium chloride (CaCl_2_) to obtain a 5 U/mL thrombin solution. For cell-fibrin-PU composites, 1 × 10^6^ hASCs were resuspended in 20 µL sterile-filtered fibrinogen solution and carefully mixed with the same volume of thrombin solution. Subsequently, the 40 µL cell-fibrin suspension was injected into one PU scaffold until it was completely absorbed by the PU foam. Afterwards, the cell-fibrin-PU composites were allowed to gel for 30 min at 37 °C. 

#### 2.1.3. Scanning Electron Microscopy (SEM)

SEM was used to visualize the surface of the PU-fibrin scaffold, the surface of the whole construct, and the surface after dividing the constructs into halves after the cell seeding procedure. Specimens were prepared as described previously [32]. Briefly, seeded PU foams were stored in a 6.25% glutaraldehyde phosphate-buffered solution. After washing procedures using Soerensen phosphate buffer (50 mM, ph7.4), dehydration in an increasing acetone series followed. The specimens were critical point dried (CPD 030; Balzers, Liechtenstein), sputtered with gold-palladium (SCD 005; Balzers), and stored in the dehydrator until examination with the scanning electron microscope (Zeiss DSM 962, Oberkochen, Germany). The analysis was performed at the Division of Electron Microscopy, Theodor-Boveri-Institute, University of Wuerzburg (Prof. Dr. G. Krohne). 

#### 2.1.4. Chondrogenic Differentiation of PU-Fibrin Composites

After polymerization, chondrogenic differentiation medium, which consisted of DMEM supplemented with 1% P/S, 100 nM dexamethasone, 100 µg/mL sodium pyruvate (Sigma-Aldrich), 50 µg/mL ascorbate-2-phosphate (Sigma-Aldrich), 40 µg/mL proline (Sigma-Aldrich), and 1% ITS-plus liquid media supplement (Sigma-Aldrich), was added to the cell-seeded PU-fibrin constructs. Expansion medium (EM-DMEM) was used for the control group:

1. Group (DMEM): Control group maintained in expansion medium.

To induce chondrogenic differentiation, the following growth factor combinations were added to the chondrogenic differentiation medium (based on Hennig et al. [25]):

2. Group (BT): 50 ng/mL TGF-ß3 and 500 ng/mL BMP-6 (PromoCell GmbH, Heidelberg, Germany).
3. Group (BBT): 500 ng/mL BMP-6 for 7 days, followed by 50 ng/mL TGF-ß3 and 500 ng/mL BMP-6.
4. Group (TI): 50 ng/mL TGF-ß3 and 100 ng/mL IGF-1 (Sigma-Aldrich).

The media of all groups were changed every second day during the culture period. Constructs were harvested after 21 days for cryosections and after 14 and 21 days for gene expression analyses using real-time polymerase chain reaction (RT-PCR).

#### 2.1.5. Histology and Immunohistochemistry

The constructs were harvested after 21 days, rinsed twice with PBS, and fixed with 4% paraformaldehyde (PFA, Serva Feinbiochemica, Heidelberg, Germany) at 4 °C for 12 h. After removing the PFA and washing the constructs with PBS, a 30% glucose solution (Merck, Darmstadt, Germany) was added for 24 h. To obtain a complete penetration of the embedding material into the PU construct, incubation with a mixture of 2.5 mL 30% glucose and 2.5 mL Tissue-Tek^®^ O.C.T™ Compound (Optimal Cutting Temperature Paraffin; Sakura, Alphen aan den Rijn, Netherlands) for 7 days under continuous shaking and room temperature followed. After 7 more days in Tissue-Tek^®^ under continuous shaking, the constructs were frozen at −25 °C for cryostat sectioning (Kryotom LEICA CM1510S, Leica Microsystems CMS GmbH, Wetzlar, Germany). 

To detect negatively charged sulfated proteoglycans in chondrogenic differentiated PU-fibrin composites, sections were stained with Alcian Blue [41] and counterstained with Nuclear Fast Red. 

The deposition of aggrecan (AGG) and collagen II (COL2) was identified by immunohistochemistry. Therefore, the labelled biotin-streptavidin method was used. Primary antibodies were purchased from EMD Millipore Corporation, Billerica, MA, USA (anti-COL2, mouse-monoclonal) and Novus Biological, Littleton, CO, USA (anti-AGG, mouse-monoclonal). Secondary antibodies (biotin anti-mouse and anti-rabbit) were purchased from Sigma-Aldrich (Steinheim, Germany). 

After fixation, the sections were blocked with a 10% solution of FCS in PBS. The addition of 3% H_2_O_2_ blocks endogenous peroxidases. After incubation of the sections with the primary antibodies and a washing step, the biotinylated secondary antibody was added. Subsequently, after washing, the sections were treated with streptavidin peroxidase (Sigma-Aldrich, Steinheim, Germany). After incubation and a further washing step, sections were covered with DAB (3′3-diaminobenzidine (DAB) enhanced liquid system, Sigma-Aldrich, Steinheim, Germany). After washing with distilled water, the sections that were treated with anti-collagen II and anti-aggrecan antibodies were counterstained with nuclear fast green (Sigma-Aldrich, Steinheim, Germany). 

Sections of human nasal septal cartilage, which remained after elective septal surgery, were used as positive controls for histology and immunohistochemistry analyses. The photomicrography and documentation were done with a Leica DMI 4000B inverted microscope.

#### 2.1.6. Real-Time Polymerase Chain Reaction (RT-PCR)

For RT-PCR analyses, PU-fibrin composites were prepared in triplicates for each patient, each growth factor combination, and each time point. On days 0, 14, and 21, PU-fibrin composites were harvested, rinsed twice with PBS, and finely minced. Total RNA of PU-fibrin composites were extracted using the RNeasy Mini Kit (Qiagen, Hilden, Germany). Reverse transcription was performed using the High Capacity RNA-to-cDNA Master Mix (Applied Biosystems, Darmstadt, Germany). RT-PCR was performed in triplicate using a cDNA input equivalent of 50 ng RNA per replicate on an RT-PCR device (Applied Biosystems, Darmstadt, Germany) using standard Taqman^®^ Gene Expression Assays and specific primer sequences for collagen II, aggrecan, and SOX-9. Relative quantification was performed and presented as values (∆∆CT values) normalised to the gene expression of the housekeeping gene GAPDH and day 0. 

### 2.2. In Vivo Studies (New Zealand White Rabbits)

**Figure 1:**
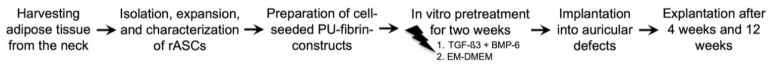
Procedure of the in vivo studies with New Zealand White Rabbits.

Animal studies were approved by the Government of Unterfranken (#55.2-2531.01-67/09, 1 December 2009). In this study, six female adult New Zealand White Rabbits weighing 3 to 4 kg were used for the experiments. Anesthesia was induced by the intramuscular injection of a mixture of 5 mg/kg xylazine 2% and 35 mg/kg ketamine. All operation procedures were performed under sterile conditions. Three procedures were performed on each animal: 1. harvesting of adipose tissue from the neck to isolate autologous ASCs 2. implantation of autologous cell-seeded PU-fibrin composites, which were maintained in EM-DMEM two weeks before implantation (group 1, undifferentiated, UD) or predifferentiated (predifferentiated, PD) in vitro with chondrogenic differentiation medium plus BMP-6 and TGF-ß3 (group 2), and 3. explantation of the constructs after 4 and 12 weeks (Figure 1).

#### 2.2.1. Harvesting of Adipose Tissue 

The rabbits were shaved on the neck under general anaesthesia and the skin was disinfected with 70% ethanol. After a skin incision, the subcutaneous fat tissue was removed under sterile conditions and transferred to a sterile tube with PBS and 1% P/S. The wound was closed with absorbable suture material. The ASCs of the rabbits were isolated, expanded, and stored according to the protocol for the hASCs described above. Instead of FCS in the expansion medium, rabbit serum (PAA Laboratories GmbH, Pasching, Austria) was used for r (rabbit) ASCs expansion. Rabbit ASCs of passage 2 were used for the implantation procedures. 

#### 2.2.2. Implantation of the PU-Fibrin Composites

PU-fibrin composites were prepared as described above and seeded with 1 × 10^6^ autologous rASCs each. Afterwards, the constructs of the first group were predifferentiated (PD) in chondrogenic differentiation medium with 500 ng/mL BMP-6 for 7 days followed by 50 ng/mL TGF-ß3 and 500 ng/mL BMP-6 for 7 days in vitro. The constructs of the second group were maintained in EM-DMEM for two weeks before implantation. The implantation procedure was performed under general anaesthesia and based on the description of the “pinna punch hole” model by ten Koppel et al. [42]. Therefore, the auricle was shaved and disinfected with 70% ethanol. The skin of the concave side of the auricle was incised and bluntly lifted off using an elevator by Plester. Carefully, as not to damage the skin of the contralateral convex side, six punch holes (biopsy punch device, Harris-Uni-Core™, Redding, CA, USA), 4 mm in diameter, were placed on each pinna. After implanting the constructs, the skin was closed with absorbable sutures and disinfected again. 

#### 2.2.3. Explantation of the PU-Fibrin Composites

The explantation procedure was performed under general anaesthesia 4 and 12 weeks after the implantation of the constructs. The operation site was disinfected with 70% ethanol, and the scar was reopened. After the skin was again carefully elevated, the implants were excised, followed by skin closure using absorbable sutures and disinfection.

After each surgical procedure, the general condition and wound healing situation of the animals were checked daily until the wounds were completely healed. 

#### 2.2.4. Processing of the In Vivo Samples

The PU-fibrin constructs were removed with adjacent cartilage and processed for histologic analyses. Fixation with 4% PFA and embedding in TissueTec^®^ O.C.T™ Compound was performed as described above. After cryosectioning the constructs into 10 µm thick sections, Alcian Blue and von Kossa staining was performed. In addition, the gene expression of SOX-9; collagen I, II, and X; and RUNX-2 was analysed using RT-PCR as described above. Relative quantification was performed and presented as values (∆CT values) normalised to the gene expression of the housekeeping gene GAPDH. 

### 2.3. Statistical Analyses

All graphs and statistical analyses were performed in GraphPad 9 (Graphpad Software, La Jolla, CA, USA). One-way analysis of variance (ANOVA) with a Bonferroni test was used to compare the means of gene expression. Significance was assumed for *p* < 0.05 and indicated in the figures. Results were charted using boxplots. Each box indicates the median, the 1st quartile, and the 3rd quartile, and the whiskers represent the minimum and maximum values.

## 3. Results

### 3.1. In Vitro Studies

#### 3.1.1. Histology and Immunohistochemistry

**Figure 2:**
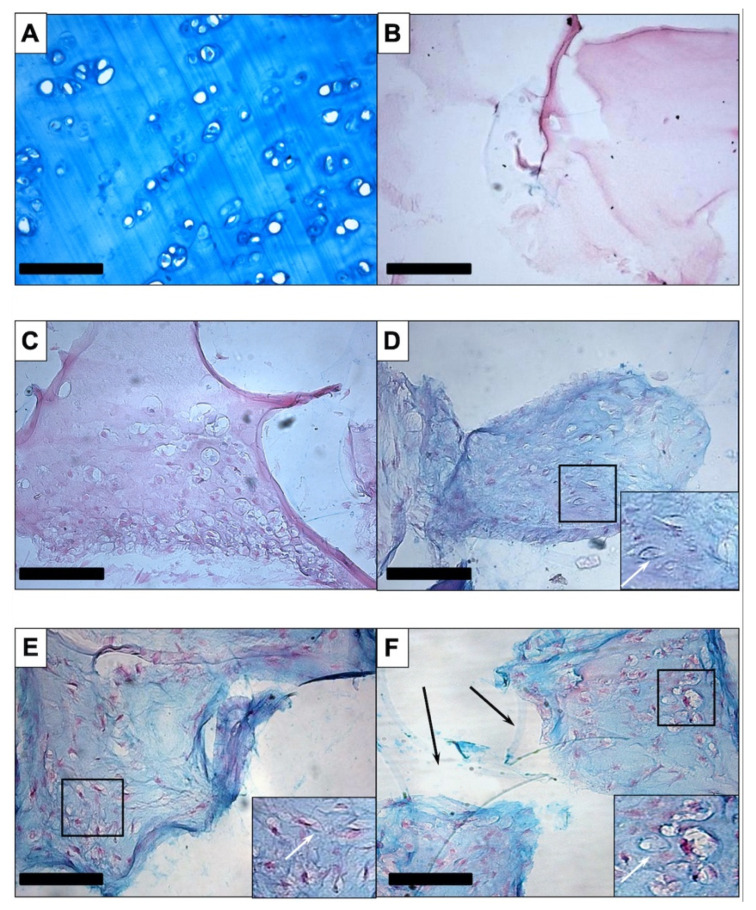
Alcian blue staining: Intense staining was seen in human septal cartilage, the positive control (**A**). PU-fibrin scaffolds without cells (**B**) and cell-seeded scaffolds maintained in EM-DMEM (**C**) revealed no blue staining. Chondrogenic differentiation in polyurethane-fibrin composites was verified by the detection of acid glycosaminoglycans in the extracellular matrix, apparent as extracellular blue-turquoise colouration in the growth factor-treated groups BBT (**D**), BT (**E**), and TI (**F**). Another interesting finding was scattered cartilage lacunae-like structures in the sections of chondrogenic-induced ASC-seeded PU fibrin constructs. Corresponding marked areas were shown enlarged in inserted smaller pictures.The white arrows highlight enhanced staining in the marginal area of the structures. Polygonal, clear sections of the polyurethane foam are highlighted by black arrows in 2F. Magnification is ×200 in all figures; the scale bar represents 100 µm.

Histological and immunohistochemical analyses were performed for qualitative assessment of the constructs. To detect extracellular glycosaminoglycans in chondrogenic differentiated PU-fibrin composites, sections were stained with Alcian Blue and counterstained with Nuclear Fast Red. Hyaline cartilage from the nasal septum, which served as a positive control, showed a uniform blue-turquoise staining of the extracellular matrix (Figure 2A). PU-fibrin composites without hASCs (Figure 2B) and cell-seeded constructs maintained in EM-DMEM showed no blue staining (Figure 2C), whereas cell-seeded PU-fibrin constructs maintained in chondrogenic differentiation medium with the growth factor combinations BBT (Figure 2D), BT (Figure 2E), and TI (Figure 2F) were typically blue-stained. In addition, the sections of the chondrogenic-induced groups revealed cartilage lacunae-like structures with accentuated staining around the lacunae (Figure 2D–F). No qualitative differences were detected between the three groups after analysing the histologic images. In Figure 2F polygonal, clear sections of the PU foam are visible. 

**Figure 3:**
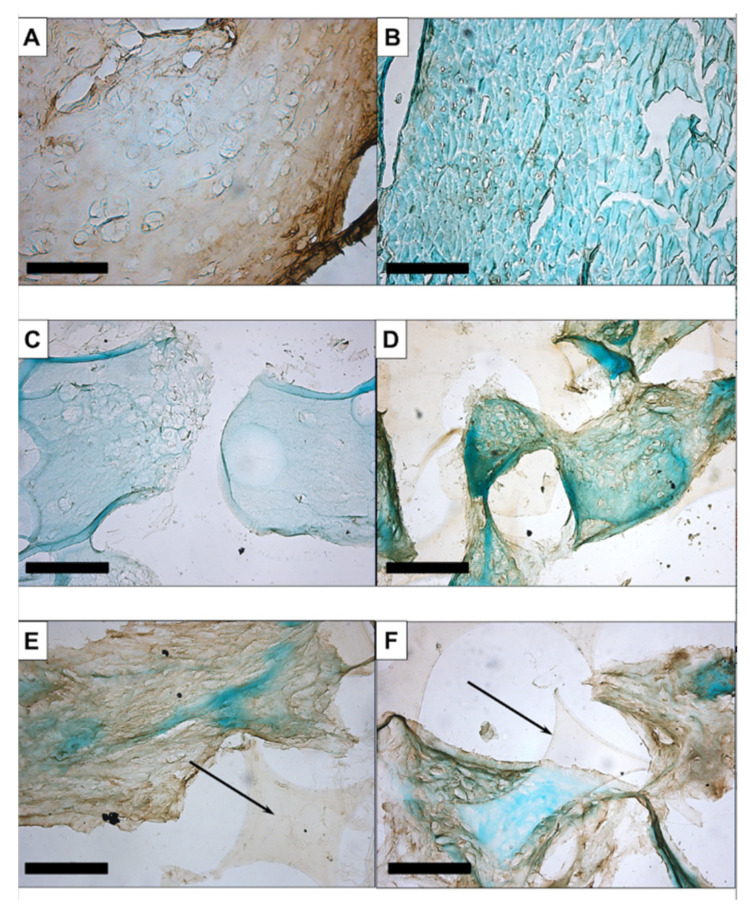
Immunohistochemistry with anti-collagen II: An even brown staining of the extracellular matrix of human septal cartilage resulted (**A**), whereas the negative control, muscular tissue (**B**), and the cell-seeded PU-fibrin construct maintained in EM-DMEM showed no brown colouration (**C**). The treated groups revealed weak staining in the BBT-treated group (**D**) and visible brown staining in the BT (**E**) and TI (**F**) group. Magnification is ×200 in all figures; the scale bar represents 100 µm.

Immunohistochemistry using anti-collagen II antibody resulted in an even brown staining of the extracellular matrix of human septal cartilage (Figure 3A), whereas the negative control, muscular tissue (Figure 3B), and cell-seeded PU-fibrin construct maintained in DMEM showed no brown colouring (Figure 3C). The treated groups showed some differences, with visible brown staining detected in the BT (Figure 3E) and TI (Figure 3F) groups. This was less noticeable in the BBT-treated group (Figure 3D). 

**Figure 4:**
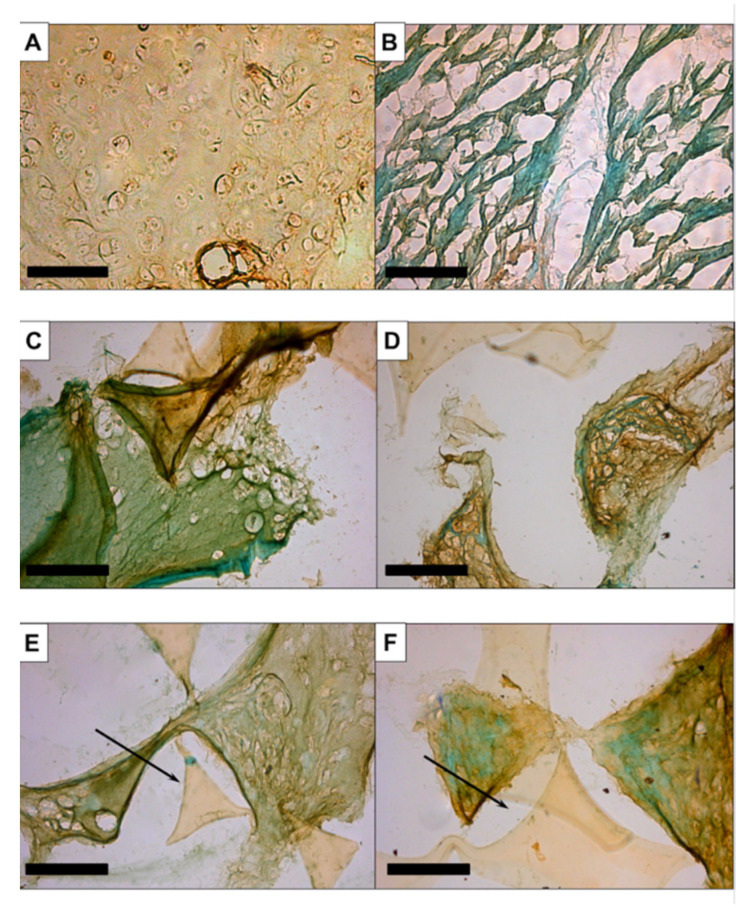
Immunohistochemistry with anti-aggrecan: There was clear staining in the septal cartilage (**A**) and no staining of the muscular tissue (**B**) or the constructs maintained in EM-DMEM (**C**). Brown staining was apparent in the treated groups (**D**–**F**). Magnification is ×200 in all figures; the scale bar represents 100 µm.

The immunohistochemical staining of aggrecan was clear in the septal cartilage (Figure 4A). No staining was seen in the muscle tissue (Figure 4B) or in the constructs maintained in EM-DMEM (Figure 4C). Brown staining was visible in the treated groups (Figure 4D–F).

#### 3.1.2. RT-PCR

**Figure 5:**
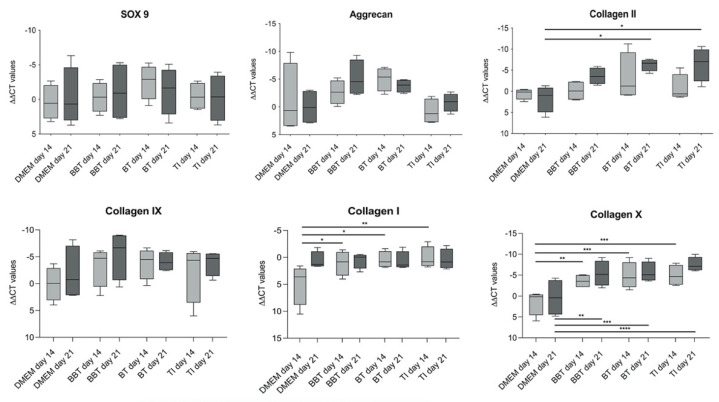
RT-PCR analyses of the in vitro PU-fibrin constructs: Relative quantification was performed and is presented as values (∆∆CT values) normalised to the gene expression of the housekeeping gene GAPDH and day 0. SOX-9: No significant differences during the time course and between the different groups. Aggrecan: No significant difference during the time course compared to the control, but a tendency towards increased gene expression in the BBT and BT groups. Collagen II: Significant increase in the BT and TI groups compared to the control after 21 days. Collagen IX: A tendency to increase was also detectable, but no significant difference was evident. Collagen I: After an initial increase compared with the control, no relevant change of gene expression was determined in any group compared to the control group after 21 days. Collagen X: Significant increase in the growth factor groups. Box-Whisker plots show the median, 1st quartile, 3rd quartile as well as minimum and maximum values of ∆∆CT, significance is indicated by asterisks (* *p* < 0.05, ** *p* < 0.01, *** *p* < 0.001, **** *p* < 0.0001).

On day 0, day 14, and day 21 of culture, samples were processed for RT-PCR to analyze the gene expression of aggrecan, SOX-9, and collagen, I, II, IX, and X normalised to the housekeeping gene GAPDH and the CT values of day 0 (Figure 5). The gene expression of SOX-9 showed no significant differences during the time course and between the different groups. The gene expression of aggrecan showed a tendency to increase in the BT and BBT group compared with the constructs maintained in EM-DMEM and the TI group; however, the increase was not significant. Collagen II, another mature chondrogenic marker, was significantly increased after 21 days in the BT and TI groups compared with the EM-DMEM group. Collagen IX gene expression also showed an increase compared with the controls on day 14 and day 21, but the difference was not significant. The expression of the dedifferentiation markers collagen I and collagen X was also examined and there was no significant difference in collagen I expression in all growth factor groups over time compared to day 0 and after 21 days compared to the control group. Collagen X gene expression was already significantly increased in the growth factor groups after 14 days compared with the control group.

#### 3.1.3. Scanning Electron Microscopy (SEM)

**Figure 6:**
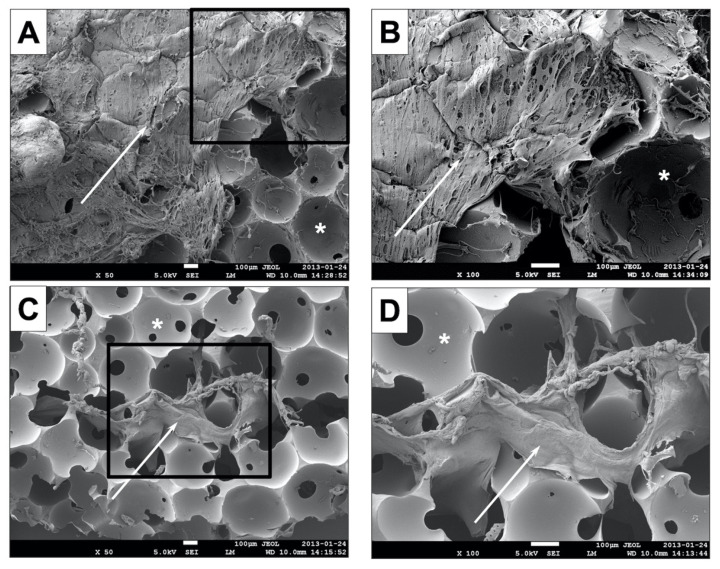
Scanning electron microscopy (SEM): The SEM shows that the cell-seeded fibrin hydrogel did not permeate the centre of the polyurethane foam. While the surface of the construct and the pores of the outer part of the PU foam were subtotally covered with the fibrin hydrogel (arrows) ((**A**,**B**), which is an enlarged detail of (**A**)), the centre ((**C**,**D**), which is an enlarged detail of (**C**)) was almost completely empty. Pores of the polyurethane foam are highlighted with asterisks. The scale bar of each pattern represents 100 µm.

SEM examination showed that the cell-seeded fibrin hydrogel did not penetrate into the centre of the PU foam. While the surface of the construct and the pores of the outer part of the PU foam were subtotally covered with the fibrin hydrogel (Figure 6A, B), the centre of the construct (Figure 6C, D) is almost completely empty. Only isolated areas with fibrin gel could be detected.

### 3.2. In Vivo Experiments in New Zealand White Rabbits

#### 3.2.1. Histology 

**Figure 7:**
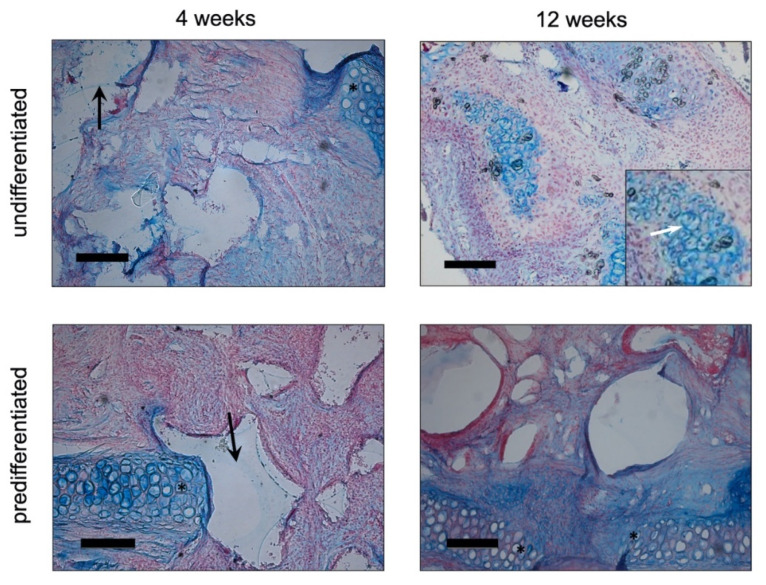
Alcian blue staining of cell-seeded PU-fibrin composites in vivo (New Zealand White Rabbit): In the Alcian Blue images, the native auricular cartilage in the region of the defect margin is visible (*). The PU-fibrin construct was slightly thicker than the native cartilage of the auricle, and thus it protruded above the level of the cartilage. Polygonally shaped sections of the PU foam can be seen in the images of the constructs explanted after 4 weeks (black arrows). After 4 weeks, an even distribution of cells and blue-turquoise stained areas could be found within the pores of the PU foam. After 3 months, there were structures with more pronounced blue colouration inside the pores of the construct similar to cartilage lacunae in both groups. The insert shows a clearly accentuated blue staining around these “lacunae” at a higher magnification + highlighted by a white arrow. Qualitatively, no remarkable difference between the groups could be detected. Magnification is × 100 in all figures; the scale bar represents 200 µm.

**Figure 8:**
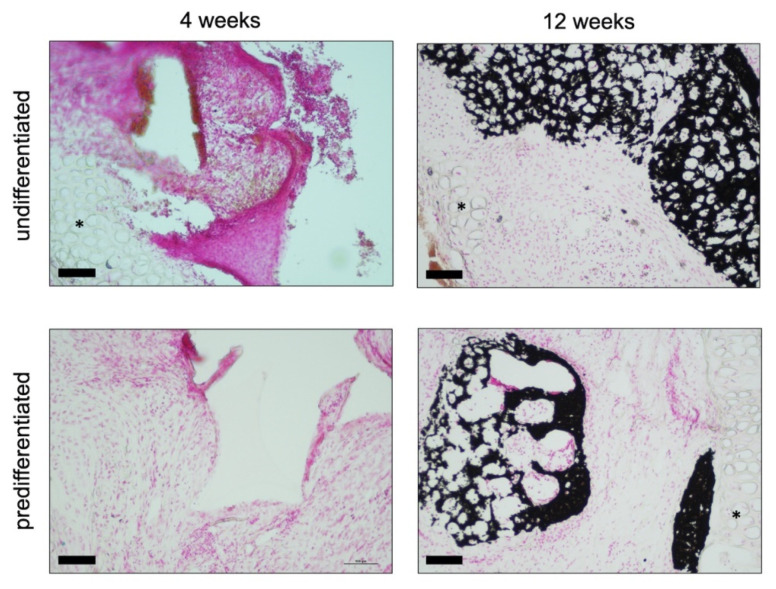
Von Kossa staining of cell-seeded PU-fibrin composites in vivo (New Zealand White Rabbit): The native auricular cartilage in the region of the defect margin is highlighted with asterisks (*). After 4 weeks, even cell distribution could be seen within the pores of the construct, stained by Nuclear Fast Red. There was no evidence of extracellular calcium deposition. After 12 weeks, distinct black-coloured areas could be detected within the constructs. There was no remarkable difference between both groups. Magnification is ×200 in all figures; the scale bar represents 100 µm.

The removed constructs were analysed using Alcian blue (Figure 7) and von Kossa (Figure 8) staining. In the Alcian Blue images, the native auricular cartilage with the typical blue staining was visible. The images showed the region of the defect margin. As the cell-seeded PU-fibrin construct was slightly thicker than the native cartilage of the auricle, it protruded above the level of the cartilage. Polygonally shaped sections of the PU foam can be seen in the images (Figure 7). Inside the pores of the PU foam, the fibrin gel with an even distribution of cells and blue-turquoise stained areas could be detected. Qualitatively, there was no clear difference between the group predifferentiated with chondrogenic differentiation medium and growth factors and the group maintained in EM-DMEM. After 3 months, the blue colouration inside the pores of the PU-fibrin construct was more pronounced, with structures similar to cartilage lacunae. Around these “lacunae”, the blue staining was clearly accentuated (Figure 7). 

Extracellular calcium deposits are shown as black nodules or areas with the von Kossa stain. There was no evidence of extracellular calcium deposition in the images showing the polyurethane fibrin constructs four weeks after implantation. However, after 12 weeks, distinct black-coloured areas could be detected within the constructs (Figure 8).

#### 3.2.2. RT-PCR Analyses

**Figure 9:**
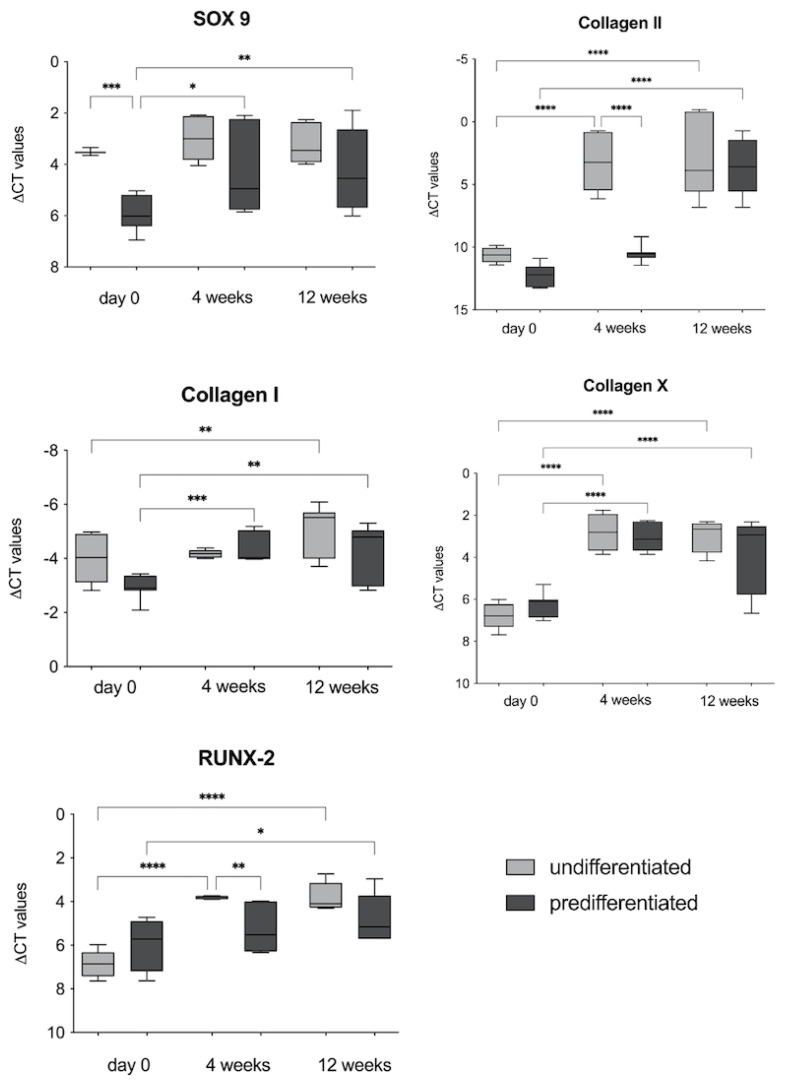
RT-PCR analysis of cell-seeded PU-fibrin composites in vivo (New Zealand White Rabbit): The gene expression of SOX-9; collagen I, II, and X; and RUNX-2 was analysed and presented as values (∆CT values) normalised to the gene expression of the housekeeping gene GAPDH 4 weeks and 12 weeks after the implantation of the cell-seeded constructs. The chondrogenic marker genes SOX-9 and collagen II were elevated in both groups after 4 and 12 weeks. After implantation, the increase in SOX-9 expression compared to day 0 was only significant for the constructs predifferentiated with BMP-6 and TGF-ß3. Collagen II expression showed a significant increase at 4 and 12 weeks compared to day 0 in the undifferentiated group (EM-DMEM) and at 12 weeks in the predifferentiated group. The expression of the dedifferentiation markers collagen I and collagen X was significantly increased for both groups over the 12 weeks in vivo. RUNX-2, a transcription factor for osteogenic differentiation, was also significantly expressed by both groups during the time course compared to day 0. Box-Whisker plots show the median, 1st quartile, 3rd quartile, and the minimum and maximum values of ∆CT; significance is indicated by asterisks (* *p* < 0.05, ** *p* < 0.01, *** *p* < 0.001, **** *p* < 0.0001).

The gene expression of SOX-9; collagen I, II, and X; and RUNX-2 was analysed 4 weeks and 12 weeks after the implantation of the cell-seeded constructs (Figure 9). 

For the chondrogenic marker genes SOX-9 and collagen II, both groups showed an increase after 4 and 12 weeks. In the gene expression of the transcription factor SOX-9, a significant difference between the groups pre-cultured in vitro was already noticeable before implantation, with a significantly higher expression of SOX-9 in the group of constructs pre-cultured with EM-DMEM. After implantation, the increase in SOX-9 expression compared to day 0 was therefore only significant for the constructs predifferentiated with BMP-6 and TGF-ß3. Collagen II expression showed a significant increase at 4 and 12 weeks compared to day 0 in the undifferentiated group (EM-DMEM) and at 12 weeks in the predifferentiated group.

The expression of the dedifferentiation markers collagen I and collagen X was also analysed, and there was a significant increase in the expression of both marker genes for both groups over the 12 weeks in vivo. RUNX-2, a transcription factor for osteogenic differentiation, was also significantly expressed by both groups during the time course compared to day 0 (Figure 9).

## 4. Discussion

Subcutaneous adipose tissue is an easily accessible and valuable source for multipotent stromal cells [22,43,44]. Mature adipocytes can be separated from the stromal vascular fraction (SVF) via enzymatic or mechanical digestion [45]. The SVF is a heterogeneous mixture of cells and a rich source for so-called adipose tissue-derived stromal cells (ASCs), which adhere to the plastic after seeding the SVF cells into tissue culture flasks [43,44]. ASCs are a subtype of mesenchymal stem cells and share their characteristic properties [22,43,44]. This makes ASCs and SVF cells valuable tools for cell-based approaches in vitro and in vivo, as has been demonstrated in numerous clinical studies in recent years, using ASCs in an autologous way as well as an allogenic one [43,44,45,46,47,48]. Automated and standardised systems for obtaining SVF in the clinical setting have facilitated cell-based therapeutic approaches [45]. The beneficial effects of ASCs are known in tissue regeneration, such as the promotion of wound healing, the prevention and treatment of scars, and the treatment of chronic inflammation [44,45,49]. Furthermore, the addition of ASCs or SVF to fat grafts leads to an increase in the volume stability and survival of the fat grafts, which is beneficial in plastic and reconstructive surgery approaches [46,50,51]. This effect of ASCs and SVF is due to their stimulation of neo-vascularization and their antiapoptotic and immunomodulatory effect by the paracrine secretion of various trophic factors, such as growth factors and cytokines [44,46,50,51,52].

The present study focused on another property of ASCs, their multi-lineage differentiation potential. The capacity of ASCs to differentiate into the chondrogenic lineage is lower than that of BMSCs [25,26,27]. Various growth factors and growth factor combinations, including members of the TGF-ß family, as well as platelet-rich plasma (PRP) as an autologous growth factor source [44,53,54], are described in the literature to effectively stimulate ASCs towards a chondrogenic differentiation capacity similar to BMSCs [25,27,29,30,31,32,55]. In the present in vitro study, different growth factor combinations containing TGF-ß3, BMP-6, and IGF, which are reported to have a beneficial effect on the chondrogenic differentiation potential of ASCs, were assessed.

A combination of PU foam and fibrin gel was used as a three-dimensional scaffold for the ASCs. The PU foam has high mechanical stability, which can prevent a deformation of the implant when used in vivo. In addition, different shapes can be produced, which allows the scaffold material to be adapted as needed. For these reasons, PU is a suitable scaffold material for cartilage tissue engineering for cartilage replacement procedures in the head and neck region, such as auricular reconstruction [5]. The combination of PU foam with a long-term stable fibrin gel was reported to ensure high cell seeding efficiency [38], even cell distribution, and homogeneous retention of the extracellular matrix (ECM) within the construct [34,36,38]. This is in contrast to the observations in the present study. SEM examinations revealed that there was less fibrin hydrogel within the central pores of the PU-fibrin construct, whereas the surface of the construct and the pores of the outer part of the PU foam were subtotally covered with the fibrin hydrogel. Constructs cut in half showed many empty pores in the central area (Figure 6). This may be due to insufficient distribution of the fibrin hydrogel within the PU foam during the preparation procedure and may be an explanation for the experience that it was very difficult to avoid the rupture of the construct during cryosectioning despite a time-consuming, repeatedly tested and modified embedding procedure. Although the PU-fibrin constructs were prepared as described by Eyrich et al. [36], who did not find any limitations in the seeding of the constructs, an optimization of the fibrin hydrogel distribution within the PU foam is required. 

Our histological and immunohistochemical data showed no clear qualitative differences in the deposition of ECM proteins between the growth factor groups (Figure 2, Figure 3 and Figure 4). In addition, gene expression analyses of the constructs after 14 and 21 days of culture were performed and compared to day 0. 

The expression of the transcription factor SOX-9, which has a regulatory role during chondrogenesis [31], showed no significant difference during the time course. Collagen IX gene expression also showed no significant difference compared to the control. There was an increase in gene expression of extracellular matrix proteins, such as collagen II and aggrecan, of the constructs treated with chondrogenic differentiation medium and growth factors; however, the increase was only significant for the collagen II expression in the BT and TI groups after 21 days (Figure 5). These results are consistent with the results of others, who described a stimulating effect on the chondrogenic differentiation of ASCs using various growth factor combinations [25,27,29,30,31,32,55]. Therani et al. [31] recently published a study on chondrogenic differentiation of human scalp ASCs using TGF-ß3 and BMP-6 and described effective chondrogenic differentiation with an increase in gene expression of SOX-9, aggrecan, and collagen II over a 14-day experimental period. A striking observation was the minimal change in SOX-9 gene expression over time, which did not correlate with collagen II expression. This is consistent with previous studies by our study group [28], but it is in contrast to others who have found a relationship between SOX-9 gene expression and collagen II using BMSCs [56]. The expression of the dedifferentiation markers collagen I, a negative marker for hyaline cartilage, and collagen X was also analysed. After an initial increase compared to the control group, there was no significant elevation of collagen I expression between the groups after 21 days. The difference at the time point on day 14 may be due to the low gene expression measured in the control group. During the time course, collagen I expression was stable and not significantly changed in the growth factor groups compared to day 0. Collagen X gene expression was significantly increased in all growth factor groups. Collagen X, a marker of hypertrophy, is an early indicator of hyaline cartilage formed during endochondral ossification and mineralization [27,30]. Ude et al. [27] evaluated cartilage differentiation of ASCs and BMSCs with TGF-ß3 alone and with the combination of TGF-ß3 and BMP-6. They found more effective stimulation of chondrogenic differentiation of ASCs by the growth factor combination. However, ASCs treated with the growth factor combination showed an increase in collagen I expression, which is not consistent with the observations in the present study, in which the collagen I expression was significantly changed in the growth factor groups compared to day 0. Collagen X expression was also increased, which the authors attributed to the two-dimensional monolayer culture in which the cells were induced [27]. Huang et al. [30] examined the chondrogenic differentiation of human ASCs in chitosan scaffolds using TGF-ß3 and BMP-6. The authors found that the combination of these two growth factors resulted in a reduction of collagen I and X expression of ASC-seeded chitosan constructs. While TGF-ß3 alone leads to endochondral cartilage formation and development towards ossification, this process can be modulated by the addition of BMP-6. Thus, the authors stated that for the formation of stable hyaline cartilage, two morphogens of the TGF-ß family are required [30]. In contrast to the observations of Huang et al. [30], collagen X was also increased in the TGF-ß3 and BMP-6 groups in the present study. This observation could be due to the different scaffold materials used for the studies, as Huang et al. suggested from their results a chondrogenic inducing effect on ASCs for the chitosan scaffold alone [30]. This effect has also been described for collagen I as scaffold material for chondrogenic differentiation, which reportedly mimics the extracellular matrix structure and promotes the spontaneous differentiation of ASCs [44,53,54]. The insufficient distribution of cell-seeded fibrin hydrogel within the PU foam described above may lead to a reduced chondrogenic differentiation capacity of ASCs due to a two-dimensional cell spreading at the pore walls [36]. Since a three-dimensional structure of the scaffold material is important to support differentiation and tissue growth [34,44,53,54], this two-dimensional distribution of ASCs on pore walls results in dedifferentiation of cells over time, which in turn may explain the increased expression of the dedifferentiation marker collagen X in the present study. An examination of the distinct areas of the PU constructs with RT-PCR and immunohistochemistry was not performed in the present study, but this is of interest for future studies to clarify the possible relationship between poor hydrogel distribution and the dedifferentiation of the ASCs. In addition, the cell distribution within the PU foam should be evaluated. 

Clear differences between the growth factor combination groups in the gene expression of the different marker genes could not be detected in the present study.

In the second part of the experiments, the behaviour of the constructs after being precultured in either EM-DMEM or chondrogenic differentiation medium with BMP-6 and TGF-ß3 was studied in New Zealand White Rabbits. The constructs were inserted into cartilage defects of the auricle according to the “pinna punch hole” model by ten Koppel et al. [42]. After 4 and 12 weeks, the constructs were explanted and analysed using histological staining with Alcian Blue and von Kossa as well as RT-PCR. 

The in vivo constructs showed a uniform distribution of the cell-seeded fibrin hydrogel within the pores of the PU foam with deposition of extracellular cartilage matrix over the time course. It is supposed that the in vivo situation leads to an integration of the whole construct into the environment, vascular ingrowth, and improved integrity of the implant.

After 12 weeks in vivo, structures resembling cartilage lacunae appeared within the implants because of the production of cartilage extracellular matrix components using Alcian blue stain (Figure 7). This is a promising observation in terms of the differentiation of ASCs towards hyaline cartilage. In addition, the RT-PCR analyses showed an increase in the gene expression of specific marker genes for mature cartilage, such as SOX-9 and collagen II over 12 weeks in vivo (Figure 9).

However, it cannot be excluded that progenitor cells from the perichondrium of the remaining local cartilage are responsible for the generation of neo-cartilage within the PU-fibrin construct since there is strong evidence for the existence of multipotent progenitor cells in the perichondrium [57,58,59,60,61]. The ASCs were not labelled before implantation; thus, it is not possible to distinguish between implanted ASCs and resident cells in the present study. Further studies with the implantation of scaffold material without ASCs will certainly provide further insight regarding the involvement of resident progenitor cells. Aside from that, it is controversial whether the implanted ASCs differentiate into chondrocytes producing the ECM in the in vivo situation or whether there is an effect of the ASCs on cartilage regeneration through paracrine secretion of various factors that stimulate resident cells to regenerate the tissue [52,58,62]. For this, ectopic transplantation of the constructs without cartilage in the surrounding area as a comparison could provide further information. 

The RT-PCR analyses also revealed an increase in the expression of collagen I and collagen X, markers for dedifferentiation. This observation could also be due to the previously explained dedifferentiation of cells within the scaffolds during in vitro pre-cultivation. Furthermore, an increase in RUNX-2 gene expression was detected as an indication of endochondral ossification (Figure 9). In line with the results of the RT-PCR, the histological images showed large areas of extracellular calcium deposition in the von Kossa stain 12 weeks after implantation (Figure 8). It has been described in the literature that TGF-ß3, rather than leading to the formation of hyaline cartilage in vivo, leads to endochondral ossification [27,30] after chondrogenic induction in vitro [63]. Farrell et al., e.g., demonstrated complete bony transformation of cartilaginous pellets after 14 weeks in vivo. The route via chondrogenic priming of MSCs is therefore very promising for research groups working on regenerative approaches for bone replacement [63]. However, the combination with BMP-6 is said to overcome this effect and to promote the formation of hyaline cartilage [30,64]. By contrast, other authors predifferentiated ASCs with the growth factors TGF-ß3 and BMP-6 in vitro for 4–6 weeks to generate hypertrophic cartilage, which in turn was used to form ectopic bone tissue in vivo [65]. In the present study, ASC-seeded constructs were predifferentiated with chondrogenic differentiation medium containing TGF-ß3 and BMP-6 for only two weeks and showed evidence of endochondral ossification with appropriate changes in the expression of specific marker genes and histologic evidence of extracellular calcium deposition after 12 weeks. However, complete bony remodelling of the construct could not be detected. 

The results of the present study did not indicate a positive effect of BMP-6 on the maintenance of a hyaline cartilage structure in ASC-seeded PU-fibrin constructs. By contrast, there was ossification of the constructs in vivo, as described in the literature for the same growth factor combination and other growth factors [30,63,65]. With regard to the generation of stable hyaline cartilage using ASCs, an interesting focus is certainly on the optimization of the scaffold and the additional evaluation of alternative scaffold materials [30,44,64,66] that promote neo-cartilage formation or the development of three-dimensional constructs even without scaffold [67]. 

## 5. Conclusions

In vitro, the differentiation of human ASCs using different growth factor combinations for chondrogenic induction was investigated. There was clear evidence histologically, immunohistochemically, and molecularly of the formation of cartilage-like tissue, but with the increased expression of collagen X, there was also dedifferentiation of the cells towards hypertrophy. In vivo, tissue formation similar to hyaline cartilage was observed, but over time, the constructs followed the route of endochondral ossification. 

## Data Availability

The data presented in this study are available on request from the corresponding author.
